# Practical Researches on the Inspection and Mensuration of the Chest, Considered as Complementary of Percussion and Auscultation

**Published:** 1839-04

**Authors:** 


### Art. II.

*Recherches pratiques sur l'Inspection et la Mensuration de la Poitrine, considérées comme Moyens diagnostiques complémentaires de la Percussion et de l'Auscultation.* Par Eug. J. Woillez, m.d., Médecin de la Maison d'Aliénés de Clermont; Membre Cor. de la Soc. Méd. d'Observation de Paris, &c.—*Paris*, 1838. 8vo. pp. 496.

*Practical Researches on the Inspection and Mensuration of the Chest, considered as complementary of Percussion and Auscultation.* By E. J. Woillez, m.d.—*Paris*, 1838. 8vo. pp. 496.

This is a work of very considerable merit; and, when we regard the comparative youth of the author, it must be allowed to indicate talent of no ordinary kind. Its distinctive character is, that it seeks to establish, with equal precision, the form of the chest in the normal state,—that which arises under such aberrations as cannot be traced to the influence of disease,—and, lastly, the variations directly produced by pathological causes. By this mode of investigation, the author expects greatly to facilitate the discrimination of all changes of form originating in disease, and thus signally to improve practical diagnosis. This excellent and sound method of prosecuting his enquiry gives even the pathological division of his work (wherein alone M. Woillez has, so far as we believe, been forestalled by any,) almost the importance and interest of complete novelty; for the observations on this head made by Dupuytren, Bouillaud, even by Louis, Dr. Stokes, and others, are only particular, limited to certain morbid states, and embrace no attempt at discovering the general laws regulating the deviations to which the form of the pectoral cavity is subject. The great fault we have to find with M. Woillez, and almost all his brethren of "the School of Observation," is, that they have deduced their conclusions from too few data, which are therefore liable to be reversed by future observers who may take a more comprehensive view. The authors are, unfortunately, at least as fond of fame as of philosophy; and their impatience for notoriety makes them pluck their fruits before they are ripe. Still, as these publications all contain a certain portion of facts, at least,—and facts faithfully observed and reported,—and as the conclusions drawn from them are valuable as far as they are borne out, (surely of a very different value from the visionary and hypothetical notions promulgated by many preceding schools, which scarcely required facts at all for their construction,) we are always glad to receive and to notice them. Our readers, we trust, will make due allowance for the deficiencies just adverted to, and separate, in their estimation, the legitimate from the illegitimate conclusions, and the solid facts from both.

M. Woillez commenced his labours by the collection of 197 cases in the wards of La Pitié and the Hôtel Dieu. These were taken without selection; except in so far as the author confined his investigations to the male sex, which was done with the obvious motive of securing a more complete examination than could have been obtained among females. These subjects are divided into two classes: in the one are included such individuals as are free from symptoms depending on the thoracic organs at the time of examination, and had never previously suffered from pectoral complaints; in the other, persons who had at a former period laboured under, or were when examined labouring under, thoracic disease, or such affections of the upper abdominal viscera as might exercise an influence on the form of the thorax. From the former M. Woillez derived his materials for the determination of the physiological shape and dimensions of the chest; from the latter, his pathological inferences.

In his opening pages, M. Woillez shows that he is not free from the passion for nosologisms characteristic of the French school generally. The elasticity of the lung is styled its *concentric force;* by *eccentric force* we are to understand that by which the diaphragm and intercostal muscles augment the capacity of the chest; when fully acted on by its elasticity, the lung is said to present its *positive volume;* and, when expanded in virtue of its extensibility so as to fill one side of the chest, that organ is said to have acquired its *relative volume.* We do not, however, object to these terms, especially to the two last, as they prove evidently useful in the course of the work. The concentric force of the lung is well illustrated in the following passage:

"When a vessel full of water is turned upside down in a chemical water-trough, the liquid within, instead of pressing against the walls of the vessel, evidently inclines, by virtue of its gravity, to escape from them. But this inclination or force is insufficient to overcome the pressure of the atmosphere, and consequently the water remains in contact with the walls of the vessel. Now, each side of the chest may be accurately compared to the vessel, and the lung to the water: the only distinction is, that in the latter instance it is not gravity that tends to operate the separation, but the proper elasticity of the lung: and if in both instances we neutralize the action of atmospheric pressure by allowing air to enter between the walls of the containing body and its contents,—in other words, to enter into the spot where there exists a tendency to a vacuum,—nothing will any longer prevent the action of gravity, on the one hand, from causing the escape of the water, and the elasticity of the lung, on the other, from diminishing the size of that organ." (p. 2.)

This illustration places in a very clear point of view the difference between the abdominal and thoracic organs, as regards their relation to the walls of their respective cavities: the former press with their entire weight against the abdominal parietes; the latter have a constant tendency to escape from theirs.

On the *momentary* changes of the form of the chest occurring in health and disease, much less is told us than the rather pompous heading to a chapter perhaps entitles us to expect. It appears that the physiological changes of the thoracic walls belonging to this class,—that is, those produced by the normal actions of the contained viscera,—consist solely of a decreased depression or temporary effacement of the intercostal spaces. This only takes place, too, when the act of respiration is forced: when effected without manifest effort, those hollows always remain apparent. Their persistence in calm *inspiration* is a necessary consequence of the play of the concentric force of the lung, which acquires increased power from the dilatation of the chest caused by the preceding inspiration. From their existence during gentle *expiration*, the author infers that that movement is solely produced by the elasticity of the lungs. Respecting the momentary modifications produced by *morbid* actions of the pectoral viscera, to which class the author refers cough, dyspnœa, excessive impulse of the heart or large vessels, there is no fact of much interest recorded. As might be expected, the same disappearance of the intercostal hollows takes place in the expiratory movement of cough as in forced expiration of other kinds. It is right to state that these different temporary phenomena, which must not be confounded with the permanent changes forming the main subject of M. Woillez's book, can be conveniently observed in very thin subjects only. Their chief interest lies in the obvious enquiry they suggest, whether their prolonged or frequent occurrence may in the end produce persistent deformity.

The general form of the chest depends on the relative extent of its different diameters; but especially, according to our author, of the antero-posterior and transverse. The symmetrical conformation of parts is estimated by a comparison of the two sides in the corresponding regions. Inspection and Mensuration are the chief means of coming to an accurate decision on these points. In theory, nothing can seem more easy than those operations; but we venture to assert that, in practice, none are found more beset with difficulties. There is really scarcely any comparison to be made in this respect between them and auscultation. They require for their proper execution not only a fund of care and patience in the observer, but of intelligence and good will in the observed. The position of the patient's head, the fall of his arms, the laxity or tension of his muscles, the degree of evenness of the place on which he is stretched, will materially affect the results of the examination. In M. Woillez's nine pages on the best methods of proceeding we, consequently, do not find a word too much, or an improbable difficulty started. Like M. Louis, he recommends the inspection of the chest in three different postures of the patient: lying in bed, seated in bed, standing, or seated on a chair. Of these, without meaning to discredit the evidence afforded by the others, the last should, we conceive, be always resorted to in preference, when practicable. Comparative certainty in its results is even, *à priori*, to be looked for, from the evident less chance of malposition of the limbs or thorax through muscular contraction; but in practice we have found it incomparably superior.

Of the different possible admeasurements of the chest, that of its circumference has alone been undertaken by M. Woillez. We do not share his opinion as to the greater difficulty of some of the others because instruments are required for their performance. On the contrary, the unyielding character of an instrument saves the operator from the main source of fallacy to which he is exposed in making the circular measurement with the ordinary linen measure. M. Woillez is now, he informs us, engaged in further researches, in which the antero-posterior, transverse, and vertical diameters of the chest are his chief concern. In making his circular measurements, he employed a tailor's measure smeared with a kind of varnish, which, he states, destroys its elasticity without affecting its pliability. In its application, he recommends it to be tightened sufficiently to depress the soft parts; an injunction of which we question the propriety: for, as he admits himself, a very slight difference of pressure in the operation may seriously modify its results; and it seems particularly strange to find him enjoining pressure in a special manner in fat subjects, in whom, according to his own observation, its less or greater force may easily cause so wide a variation as half an inch in the measurement obtained. The benefit stated to be derived from this mode of proceeding is, that the results will approach more closely to what they would be if the individuals were thin. This unquestionably would be a great advantage, if the principal point were to determine and compare the absolute capacity of the entire chest in a number of subjects; but, both in reality and in M. Woillez's pages, this is a secondary object of enquiry.

Our author does not reckon the *ordinary* movements of respiration among the modifiers of the results of mensuration. In full-chested persons, he found the extent of each side increased by three centimètres* during strong inspiration; but, when this was calm, no dilatation was shown by the measure. The cause of this is not evident; unless we admit it to be the simple difficulty of estimating half of so trifling an expansion as the entire cavity undergoes. Yet nothing more satisfactory is, if we credit M. Woillez, to be learned by throwing the measure round the entire chest at once; for, in sixty cases in which he followed this, the ordinary plan, the general movement and dilatation of the thorax prevented him from making an accurate calculation of its alteration of size. Our author's sole measurement, it is important to remember, was in all instances made from the median part of the ensiform cartilage to the spinous processes of the vertebræ. This is evidently not enough, however, though M. Woillez is quite excusable for not having increased the number, as he would have thereby increased, out of all proportion, the difficulties of his analysis. A perfect estimate of the chest's dimensions should, we think, require the following measurements. Every one of them is, or may become, of importance in a diagnostic point of view.

A. General.

- *a.* Circular

  1. Midway between nipples and clavicle.

  2. On level of nipples.

  3. Opposite ensiform process.

  4. Opposite umbilicus.

- *b.* Transverse

  1. From point of one acromion to that of the other.

  2. On level of nipples.

- *c.* Antero-posterior

  1. On level of nipples.

  2. Under clavicles.

- *d.* Vertical.

B. Partial.

- *a.* Horizontal — From each nipple to median line of sternum.

- *b.* Vertical

  1. From sterno-clavicular articulation to nipple.

  2. From nipple to antero-superior spinous process of ileum.†

  3. From most dependent point of twelfth rib to same process.‡

The total number of cases analyzed by M. Woillez amounts to 197. Of these, 73 belong to the physiological order; 124 to the pathological. How these terms are to be understood we have already explained. No pains appear to have been spared by the author in investigating the previous history of his patients; and the reader may therefore, we think, rest satisfied as regards a fundamental point, the correctness of the above classification.

It is important to premise that, by a regularly formed chest, M. Woillez understands one "perfectly symmetrical in all its parts, free from partial

* The centimètre is equal to 0·393708 of an English inch.

† We have found a difference of three fourths of an inch between the sides in this measurement in cases of chronic pleurisy.

‡ In the same affection we have noted half an inch difference in this measurement.

anormal prominences or depressions, and presenting a visible excess in its transverse to its antero-posterior diameter." (p. 28.) The meaning here is fortunately more clear than the expression. By anormal (the word which causes the difficulty, and gives the whole definition the air of a truism,) M. Woillez means those elevations and depressions distinct from the well-known intercostal and sternal hollows, &c. which he calls normal and has described at length. All modifications of the regular form are styled *heteromorphisms:* these are either, from their extent, *partial* or *general.* Moreover, as deviations from the perfect type may exist where no morbid action, either in the thorax or abdomen, can be traced in their production, we have a class of *physiological* heteromorphisms; while such as have been obviously effected by diseased action within those cavities constitute the order *pathological.*

We shall introduce our analysis of the author's summary by two or three tables, from which a number of propositions are derived: they refer to the age, trades, strength, and stoutness of his patients.

1. Age.

| | | |
|---|---|---|
| 19 subjects were aged from | | 15 to 20 |
| 71 ...... | ...... | 21 to 30 |
| 44 .. ... | ...... | 31 to 40 |
| 26 ...... | ...... | 41 to 50 |
| 22 .. ... | ...... | 51 to 60 |
| 9 ...... | ...... | 61 to 70 |
| 6 ... .. | ...... | 71 to 78 |

2. Trades.

| | |
|---|---|
| Non-laborious . . . . . | 64 |
| Employing upper extremities much . | 83 |
| Requiring active exertion of the whole frame . . . . . . | 50 |

3. Strength.

| | |
|---|---|
| Robust subjects . . . . | 67 |
| Moderately strong . . . | 107 |
| Weak . . . . . . . | 23 |

4. Stoutness.

| | |
|---|---|
| Fat subjects . . . . . . | 26 |
| Moderately full in person . . | 135 |
| Spare in habit . . . . . | 36 |

A regularly formed chest, according to M. Woillez's definition, is much more rare than we should have imagined: the thorax had that character in forty-one subjects only. As no accurate investigation of the matter had ever been previously made, our erroneous impression is not to be wondered at. The comparative frequency of perfect conformation at different ages is shown in the following table:

| | | | |
|---|---|---|---|
| From æt. | 15 to 30 | 23 subjects | = $0{\cdot}25\frac{5}{9}$ |
| ...... | 31 to 40 | 9 ... | = $0{\cdot}20\frac{5}{11}$ |
| ...... | 41 to 50 | 5 ... | = $0{\cdot}19\frac{3}{13}$ |
| ...... | 51 to 60 | 4 ... | = $0{\cdot}18\frac{2}{11}$ |
| ...... | 61 to 70 | 0 | |

The decimals are calculated by comparing the number of perfectly formed individuals in each period with the total number of patients of that age; they, therefore, represent accurately the relative frequency of well-formed thoraxes among those patients. We cannot admit, however, that the number of facts here examined is sufficient to give the results from them the importance of a law; but we think we shall not widely err in affirming, on the authority of this table, that, in a series of individuals of advanced age, there will be found fewer regularly-built chests than among a like number of young subjects.

In M. Woillez's three categories of inactive occupations, of those requiring exertion of the upper extremities, and of those by which the whole frame is kept in active play, the respective numbers of well-formed chests were 16, 18, and 7: so that their relative frequency was 0·25, $0{\cdot}21\frac{57}{83}$, and 0·14. The result of observation here coincides remarkably with what might in theory have been expected. Other tables show but a very trifling difference indeed in the frequency of well-formed thoraxes in individuals varying in point of strength, height, and fulness of person. Every one of the left-handed subjects met with by our observer presented a peculiar defect, of which we shall presently give some account. The conformation of the chest is more frequently found perfect in individuals who have never suffered from pectoral complaints than in those of the opposite description. Of the 41 well-formed subjects, 26 belonged to the former class, 15 to the second. The relative majority, too, is greater than the absolute.

Of 86 physiological cases, 26, or $0{\cdot}30\frac{10}{43}$ were found perfect;

111 pathological ... 15, or $0{\cdot}13\frac{57}{111}$ ......

But this difference is nothing more than might be expected when we recollect that physiological heteromorphisms may exist in subjects affected with thoracic disease, as well as in those free from them; while the former are, in addition, exposed to the chance of pathological deformity.

The numerical method has brought to light very unlooked-for truths; but assuredly it has produced no more startling result than that with which the chapter on *physiological* heteromorphisms opens. We allude to the fact that 132* out of his 197 patients presented modifications of form belonging to this class. Of these, as we have already said, some were *partial;* others, affecting the normal ratio of the different diameters of the chest, *general.*

The partial heteromorphisms are first examined, and the author commences with such as affect the sternal region, including prominences, depressions, and deviations. Of the former he met with thirty examples; in all of which, with one exception, the prominence existed at the articulation of the two upper pieces of the sternum. These deviations of form are shown by a table not to be congenital, but developed with the progress of years. It is evident they exercise no unfavorable influence on the duration of life. However, our author's observations throw no light on the cause of their appearance: trade, strength, height, &c. possess no marked influence in this way. Depression of the sternum existed in twenty-two subjects. In two instances they were congenital, and seated at the central portion of the bone; in the rest accidental, and affecting its inferior part. The latter, so far as M. Woillez's experience goes, are not produced in the manner usually supposed, by continual pressure on the part. On the contrary, of his twenty-two cases, three only occurred in shoemakers, (though he met with twelve individuals belonging to that trade;) while, of the remaining nineteen subjects with depressed sternum two only (they were turners) were in every way exposed to pressure of that bone. Nor would it appear that there are any grounds for the current belief that this depression affects injuriously the subjacent

* This is a greater number than that of the physiological cases; the surplus is made up by such subjects affected with thoracic disease as presented heteromorphisms of exactly the same description as subjects who had never had any pectoral complaint. Their cases are, for this reason, called physiological, though ranked generally with the pathological. It must be confessed that some confusion arises from the different uses of the word physiological.

organs. In his two cases of congenital depression, no pectoral disorder had ever existed, though the patients had passed their thirtieth year. Besides, sternal depression occurs with somewhat greater frequency in his physiological cases than in the others. It has been also supposed that the heteromorphism under consideration occasions asthma. On first sight, M. Woillez's cases seem to support that opinion; for six of the twenty-two patients were subject to dyspnœa, and they were all affected with marked pulmonary emphysema. M. Woillez, however, contends, with many others, that asthma is something different from both dyspnœa and emphysema.

M. Woillez met with but one example of lateral deviation of the sternum independent of dorso-vertebral curvature. The middle line of the bone inclined in this case slightly to the right at its lower part, and the deviation had given an arched shape to the right external side of the chest. A similar phenomenon, as is well known, occurs in cases of curvature of the spine. The displacement in this subject appeared to have originated in the constant contraction of the right pectoralis major required in the exercise of his calling, that of a water-carrier.

The next class of *latero-anterior* heteromorphisms comprehends seven varieties. Foremost comes undue prominence of the left side, occurring in fifty-two subjects. This elevation, as observed by M. Woillez, cannot be the same as that described by writers as a consequence of marked right dorso-vertebral curvature; for no such deformity existed in any of those fifty-two subjects. Besides, among thirteen patients in whom the latter did exist, the heteromorphism in question was observed three times only. Considered in itself, this prominence presents no irregularity of form, and it evidently consists solely in an increased convexity of the part. It may be *general,* that is, it may occupy the whole left anterior thoracic region; or be, as the author terms it, *sterno-mammary;* in which case it is bounded by the nipple externally, and terminates above about an inch below the clavicle. Both kinds are visible on first inspection, if the subject be well placed. When either is marked, the difference of convexity between the two sides of the chest is calculated approximatively by M. Woillez at three or four lines. In fat subjects, the surface of heteromorphism is described to be uniform; in meager individuals, the natural prominence of the ribs and hollowness of the intercostal spaces are both increased, so that the characteristic unevenness of the chest is proportionally augmented. But the anterior left prominence is not in all instances produced by undue convexity of the ribs: the application of the hand shows that, in certain individuals, it depends on hypertrophy of the soft parts. The dull sound, too, elicited by percussion gives similar evidence; and, what is remarkable, this species of prominence would appear to be peculiar to *left-handed* subjects; at least, six such persons met with by our author presented a prominence in this region. In three of them it was clearly due to relative thickness of the soft parts; the fatness of the others prevented its exact character from being satisfactorily made out, but it certainly had not the manifest appearance of *costal* convexity. These facts receive corroboration from the total absence of the prominence in forty-seven right-handed patients; but we cannot think M. Woillez happy in his conjecture as to the cause of this peculiarity. He is surely hardly defensible in accounting for it by hypertrophy of the left pectoralis muscle, consequent on its great action in left-handed subjects, when he has encountered no example of similarly constituted prominence in right-handed subjects on the right side. Dissection can alone decide the question.

The cause of left anterior prominences generally is obscure. They occur at every age, in M. Woillez's tables, with nearly equal frequency, and appear uninfluenced by trades; but they are somewhat more frequent among robust, full, and tall subjects than the weak, spare, and short of stature. They cannot be said to exercise any injurious effects on the thoracic viscera; for they existed in 0·32 of the physiological cases, and only in 0·21 of the pathological. The increased convexity of the ribs forming them does not involve any diminution in their remaining length. This is shown as well by the regularity of form of other parts of the chest as by mensuration, which proves the existence of an actual expansion of the thoracic walls.

The next variety of the present class, *anterior right prominence*, is extremely rare as a physiological heteromorphism: it occurred in two only of M. Woillez's cases. The third variety, a *symmetrical prominence of the sides*, quite distinct from that produced by excessive growth of the breasts, occurred in the same number of instances. Under *irregular anterior prominences* he comprehends some very rare cases, five in number, wherein the sternal extremities of the costal cartilages were unduly elevated. When a single rib is prominent, the second is always the seat of the heteromorphism. This peculiarity occurred in twelve cases. He observed but two examples of his sixth variety, *anterior lateral depression*. On the *relative position of the nipples* he founds his seventh series. It appears that the left nipple is occasionally placed from half an inch to an inch lower than the right, in individuals who have never suffered from thoracic disease. M. Woillez observed this physiological departure from symmetry in seven subjects. In five of these it coexisted with the anterior left prominence, which helps to distinguish it from the lowered nipple of chronic pleurisy. In no instance was the right placed lower, in the physiological state, than the left nipple; which shows that, when it is so placed, it constitutes a very important sign of absorption of pleuritic effusion.

Of the physiological heteromorphisms of the dorsal region, that consisting of a general prominence on the right side is by far the most frequent. It occurred in fifty-eight subjects, or in 0·29 of the cases; and in nineteen instances coexisted with the left anterior prominence, of which we have also noted the great frequency. It seems impossible to assign right curvature of the spine of the ordinary kind, manifested externally by deviation of the spinous processes, as the cause of this prominence; for, in the fifty-eight subjects, it was detected four times only: but, as a species of curvature has been described by orthopedists dependent on *torsion* of the vertebræ, and which does not betray itself externally by any mal-direction of those processes, M. Woillez enquires whether such curvature might have existed in his cases. By a close train of induction he succeeds in showing its improbability: nevertheless, in the absence of the evidence of dissection, we share his unwillingness to make any decided affirmation on the point. It is plain that, if the dorso-lateral prominence and the torsion of the vertebræ coexisted in all cases, the former would be a more certain external sign of vertebral deformity than even the deviation of the spinous processes; whereas, the prominence would be proved independent of vertebral curvature of every species, if the torsion did not exist. This heteromorphism was more frequent in the physiological than in the pathological cases, in the ratio of 0·34 : 0·25; and cannot, therefore, be supposed to take any part in the production of thoracic disease or in shortening life.

Under the denomination of *general heteromorphisms*, M. Woillez comprises such as depended on an apparent want of regularity between the relative extent of the principal diameters of the chest. Here will be found a very interesting disquisition on the *cylindrical* thorax. The following table gives an excellent idea of the condition of the chest in the author's physiological cases, and comprises the substance of many pages:

| | | No. of Cases. |
|---|---|---|
| 1. | General prominence of the right side of the back | 58 |
| 2. | General left anterior prominence | 52 |
| 3. | Regular conformation of the chest | 41 |
| 4. | Sternal prominences | 30 |
| 5. | Sternal depressions | 22 |
| 6. | Transverse narrowness of the chest | 17 |
| 7. | Dorso-vertebral deviation | 16 |
| 8. | Double prominence of anterior part of the second ribs | 12 |
| 9. | General prominence of the left side of the back | 10 |
| 10. | Non-symmetrical position of nipples | 8 |
| 11. | Partial prominences of the right side of the back | 7 |
| 12. | Anterior prominence of one of the second ribs | 4 |
| 13. | Prominence of the cartilaginous border of the left ribs | 3 |
| 14. | Partial prominence of the left side of the back | 2 |
| 15. | Symmetrical prominence of both sides anteriorly | 2 |
| 16. | Anterior right prominence | 2 |
| 17. | Lateral deviation of sternum | 1 |
| 18. | Prominence of the sternal extremity of the cartilages of the left true ribs | 1 |
| 19. | Double symmetrical prominence of the nipples | 1 |
| 20. | Double symmetrical depression of the submammary regions | 1 |
| 21. | Non-symmetrical position of shoulders | 1 |
| 22. | External arching of right side | 1 |

The cardinal fact proved by this table is, that regular conformation holds only the third rank in frequency, even in physiological cases. As the number of heteromorphisms (251) is considerably greater than that of the subjects presenting them (135), it is clear several must have coexisted in some individuals. Such was the case in 63 out of the 135 patients: in them two, three, four, or even five existed together.

With respect to the relation subsisting between the age of subjects and the most common of the conditions enumerated above, regular form, general prominence of the right or left back, curvature of the spine, and transverse narrowness of the chest are most frequent in young subjects; whereas, the double prominence of the second ribs, and the sternal prominences and depressions, occur in larger proportion at a more advanced age.

The facts we have so far considered are those learned by *inspection:* we now proceed to the analysis of M. Woillez's chapters on the general capacity of the chest, and on the relation subsisting between the circular extent of its sides in the normal state; these are founded on the results of *mensuration*. The number of facts reviewed bearing on the first of these questions amounts to 133. The mean circular capacity equalled $82\frac{4}{13}$ centimètres; the lowest, 72; the largest, 97. The average varied in the following proportions with the age of the subjects:

| | | | |
|---|---|---|---|
| From æt. | 16 to 20 | 12 subjects, | $0{\cdot}75\frac{5}{6}$ mètre. |
| ... .. | 21 to 30 | 55 ...... | $0{\cdot}81\frac{44}{55}$ ... |
| ...... | 31 to 40 | 30 ...... | $0{\cdot}82\frac{1}{2}$ ... |
| ...... | 41 to 50 | 14 ..... | 0·84 ... |
| ...... | 51 to 60 | 17 ..... | $0{\cdot}85\frac{15}{17}$ ... |
| ...... | 61 to 78 | 5 ...... | $0{\cdot}84\frac{9}{10}$ ... |

Hence it appears that the capacity of the thorax increases regularly from puberty to the sixtieth year; the total mean enlargement during that period amounting to ten centimètres. Other tables show that its mean extent is greater in robust, fat, and tall subjects, than in those of the opposite form or constitution. This might have been foreseen: but we were unprepared for the conclusion to which we are next led, namely, that trades requiring but little muscular exertion of any kind are more favorable to development of the chest than those bringing the upper extremities into constant and severe action. We transcribe it to show that the large number of cases examined precludes the idea of mere coincidence.

| | | Average capacity of the thorax. |
|---|---|---|
| Non-laborious trades . . . . . | 46 subjects | $0{\cdot}82\frac{25}{92}$ mètre. |
| Trades requiring frequent use of upper extremities, | 54 ...... | $0{\cdot}80\frac{51}{54}$ ... |
| Trades calling for activity of the whole frame . | 33 ...... | $0{\cdot}84\frac{43}{66}$ ... |

The mean capacity of the chest varied in the strictly physiological cases, and in those in which, though ranked with the pathological, disease had not affected that capacity. The first class of sixty-six subjects gave an average of 83·4 centimètres; the second, of sixty-seven patients, a mean extent of 81·4. Now, in another part of his volume, M. Woillez has shown that, in the sixty-six former individuals, the average capacity varied between 84·4 and 81·8 centimètres; according as there existed or not physiological prominences capable of affecting the measurements. From these data he infers that the mean normal width of the thorax, when no circumstance capable of really modifying it exists, varies between 81·4 and 84·4 centimètres.

The relation subsisting in the physiological state between the circular comparative extent of the two sides of the chest, is next discussed. Symmetry of conformation, as had been shown on a small scale by other observers, is extremely rare: the right and left segments were found equal in twenty-seven only of 133 subjects. The right side was more extensive than the left in ninety-seven, and the left than the right in nine individuals. The excess of width of the right division of the chest varied from one half to five centimètres; the mean being 1·4. But, what is more remarkable, this inequality existed even in the series of thoraxes answering the description already given of perfect conformation. In thirty-six of the forty-one chests belonging to that category, the right admeasurement exceeded the left by from one to three centimètres; in five, both segments measured alike; the left consequently never exceeded the right.

From the foregoing exposition of M. Woillez's physiological facts and inferences, the reader will be enabled to judge of the stability of his conclusions respecting the pathological changes of form of the chest. whether caused by diseases of the thoracic or upper abdominal viscera. It does not enter into his plan to notice such heteromorphisms as originate in a morbid state of the walls themselves of the thorax; such as prominences produced by irregular union of fractures, tumours of the bones or of the soft parts, &c. Those to which he directs his attention are the physical effects of various diseases on the subjacent organs. They may be peculiar to those diseases, or perfectly similar to those observed in healthy subjects. And it appears, from a careful revision of his facts, that M. Woillez is correct in stating, as a general law, that the eminences and depressions of the pathological order affect a preference for such regions as are most frequently the seat of those of physiological nature. He informs us, too, as a practical point of much importance, that, in consequence of the greater normal width of the right side, morbid depressions or retractions are more easily ascertainable by mensuration when seated on that side; and, *vice versâ*, dilatations of the same kind more easily made out on the left.

We pass over the section on Bronchitis, which affection does not appear to exercise any very manifest influence on the form of the chest, and arrive at that on Pneumonia.

The reader is aware that a severe controversy formerly existed between Broussais and Laennec, respecting the possibility of dilatation of the chest as a result of hepatization of the lung; and that the point originally at issue still remains undecided. According to M. Woillez, the disagreement between observers springs from their not taking into consideration the positive volume of the lung, or its elastic force; yet, though he, of course, avails himself of his knowledge of these, he is very far, we apprehend, from removing all chance of further debate. The opinion of Laennec, who supported the negative of the question, is adopted by him on the following grounds: When the lung is hepatized in its entire extent, its elasticity is destroyed; and, as all pathologists have noticed, no contraction of its substance takes place when its chest is opened. The *positive* volume of the organ is therefore considerably increased, inasmuch as it equals the fullest *relative* volume it acquires in the healthy subject while alive. But this by no means proves that, once there is a resistance to overcome (such as that of the thoracic walls), the volume of the organ will still further increase: on the contrary, M. Woillez believes, with Laennec, that, from its diminished cohesion and loss of elasticity, it must be incapable of resisting the most trifling compressing force. There does not seem to be much more here than an application of new terms to old things well understood previously. But, be that as it will, we should have greatly preferred an inductive solution of the question from his own observation to any *à priori* attempt at its settlement, however ingenious, and however securely based in appearance on physical laws. M. Woillez is unfortunately unable to content us in this respect, as he met with but two cases of complete hepatization while engaged in the present enquiries. He informs us that a *partial* contraction, ascertainable both by inspection and mensuration, ensues at a variable period of convalescence from pneumonia; and suggests the probability of its being caused by the absorption of the slight pleuritic effusion that usually attends the parenchymatous inflammation. With respect, however, to the fact of retraction occurring in the advanced stage of this disease, M. Woillez has been anticipated by that able observer, Dr. Stokes: and the testimony of the latter gentleman goes further than our author's; for he states that he has seen contraction very strongly marked in a case of asthenic inflammation of the lung, wherein "there was not the slightest appearance of liquid effusion into the cavity of the pleura." This fact shows that M. Woillez's explanation is not applicable in all instances of the occurrence of such deformity. It is worthy of remark, too, that, in Dr. Stokes's case, the contraction "seemed to affect the whole side more than what is generally found in pleurisy."

In our recent review of the Memoirs of the Medical Society of Observation, we entered so fully into the question of the modifications of form produced by emphysema—noticing, too, in passing, some of M. Woillez's opinions, then known only through a thesis,—that our analysis of the bulky chapter on that disease in the present volume need only refer to a few of its chief points. In speaking of the physical condition of the lung in emphysema, M. Woillez states that "its elasticity is augmented in consequence of the hypertrophy of the walls of the vesicles." How shall we reconcile this opinion with the assertion of Magendie, that "emphysema is essentially characterized by want of elasticity in the lung?" The contradiction is the more strange as each bases his opinion on the same unquestionable fact, namely, that the emphysematous lung does not contract when the chest is opened after death. The opinions are indeed irreconcileable as they now stand; but M. Woillez has evidently made an improper use of the word elasticity.

In the article alluded to, we noticed the important fact of the frequency of left anterior prominence, and added a few words on its influence on the diagnostic value of such heteromorphism in emphysema. In the work before us this question is very closely examined: we extract a part of the discussion.

"General anterior prominence is infinitely more frequent at the left than the right side in emphysematous subjects. When the patients are tolerably stout in person, these prominences are identical in appearance with the analogous physiological ones already described; but, in the former case, percussion gives a clear sound, and auscultation detects a weaker murmur than at the opposite side. When, on the other hand, the intercostal spaces are visible, from the leanness of the patients, they are seen to be less hollow than elsewhere, or completely effaced at the dilated part: under these circumstances, no shadow of doubt can exist as to the pathological nature of the prominence. But, if all the sides exist as just mentioned, except that the intercostal spaces are as hollow as at the non-dilated side, the prominence cannot be considered as the result of emphysema, even though the subjacent portion of the lung be evidently affected with that disease, but as belonging to the physiological order. Such, too, is the apparent similitude existing between these two kinds of heteromorphism that, I am of opinion, we ought not to consider any prominence *incontestibly pathological*, unless the physical signs of emphysema are more strongly marked behind it than in any other part of the chest. It is true that, setting aside this last point, there could not be much doubt as to the emphysematous origin of the heteromorphism, if the intercostal spaces were less distinct there than elsewhere. Nevertheless, even in this case, we are not authorized to make a positive assertion; because it may so happen that an emphysematous lung may begin to dilate the chest in the site of a physiological prominence, and lead us to suppose the heteromorphism observed wholly emphysematous. But it may be urged, that emphysema dilates the left more easily than the right side of the thorax. I confess that I believe such to be the fact in the majority of cases; but it is not the less true that we ought still to have our doubts where the emphysematous lesion appears equally developed on both sides. There would be less room for indecision, perhaps, if the prominence were on the right side: physiological prominences in that quarter are indeed so rare, that it is probable all such heteromorphisms, provided they appear pathological, do in reality belong to that class." (p. 394.)

M. Woillez's experience confirms that of M. Louis, as to the subclavian prominence constituting an almost pathognomic sign of emphysema. In his 197 cases, it occurs only where that disease existed. With regard to the form of the posterior part of the thorax in emphysema, a point neglected by M. Louis, our author found the right side generally prominent in five, and the left in two, of the twenty-four subjects whose lungs presented that lesion: and even in these few cases the heteromorphisms were identically the same in character as those observed in the physiological state; while auscultation and percussion gave no normal result in their neighbourhood. Besides, their frequency in those subjects was not greater than in healthy individuals. We subjoin the instructive table given by our author of the different heteromorphisms observed in his emphysematous cases. The mean number for each individual is 2·4.

| 1. *Physiological Heteromorphisms.* | | 2. *Pathological Heteromorphisms.* | |
|---|---|---|---|
| Sternal depressions | 6 | Left sterno-mammary prominence | 2 |
| Prominence of right side of back | 5 | Anterior left general ditto | 3 |
| left anterior side | 4 | Left subclavicular ditto | 6 |
| sternum | 5 | Left postclavicular ditto | 5 |
| left side of back | 2 | Globular form of chest | 2 |
| Vertebral heteromorphisms | 2 | Right sterno-mammary prominence | 2 |
| Want of symmetry of nipples | 2 | Right subclavicular ditto | 1 |
| Double anterior prominence of second rib | 1 | Right postclavicular ditto | 1 |
| lateral prominence | 1 | Right and left postclavicular ditto | 1 |
| Prominence of cartilaginous border of ribs | 1 | | |

3. *Heteromorphisms of doubtful nature.*

| | |
|---|---|
| General left anterior prominence | 3 |
| Left sterno-mammary ditto | 3 |

Neither the mean nor the extremes of thoracic capacity appear much affected by the emphysematous dilatation. This is seen by the following comparison:

| | Mean capacity of chest. | Extremes of ditto. |
|---|---|---|
| In 24 emphysematous cases | 83·5 cent. | 75 and 95 cent. |
| In 133 physiological cases | 82·3 cent. | 72 and 97 cent. |

The comparative circular extent of the two sides is, however, modified. Instead of finding, as in healthy subjects, a mean excess of one centimètre and a half on the right side, he ascertained that the excess averaged in these patients only three tenths of a centimètre.

Continuing his enquiries into the influence of disease of the contained viscera on the form of the chest, M. Woillez comes to that of hypertrophous heart. He first shows, by a striking quotation from his *Traité des Maladies du Cœur*, that Senac maintained hypertrophy of the heart to be an efficient cause of permanent arching of the precordial region; and so deprives M. Bouillaud of his assumed *discovery* to the same effect. But the alleged discovery, no matter to whom it belong, is, according to M. Woillez, a mistake. He is disinclined to admit that the seven cases reported by the latter writer as examples of vaulted form of that region, brought about by the cause in question, really afford examples of such a phenomenon; because, in the first place, in three of these cases, adhesion of the right lung, or of both, was found on dissection; and, consequently, the precordial elevation may have existed simply in appearance, owing to a real depression on the other side. This seems sufficiently plausible for an hypothesis, in cases where the right lung was alone affected; but how it impugns the justness of M. Bouillaud's opinion, in the instances where both were diseased, we are at a loss to perceive. The probability that some of the remaining prominences were of physiological origin is urged; and, of course, we can find no proof to the contrary. Even admitting M. Bouillaud's notion to be correct as to the cause of the dilatations he observed, precordial elevation must be a very rare consequence of hypertrophied heart, as he only noted it in seven among fifty-four subjects labouring under that affection.

M. Woillez does not add anything to our fund of means for detecting aneurism of the aorta; but attempts to prove that the existence of a prominence in the subclavicular region (a sign of which many of our readers must, like ourselves, have felt the practical value,) is not entitled, when slightly developed, to the confidence usually given it. It is true he has shown that prominence of the second rib, as well as elevation of the upper part of the sternum, may occur in the physiological state. But the former is excessively rare, and the latter would be free from pulsatile movement; and it is surely straining a point to suppose the observer deceived into the belief of the existence of an aneurism behind the former of these physiological heteromorphisms, by the violent beating of the heart in a nervous subject.

The influence of hypertrophied liver and spleen, and of gaseous expansion of the stomach and large intestine, on the dimensions of the lower part of the chest, is considered in three separate articles. The prominence of the right hypochondrium, consequent on enlargement of the liver, must, beyond a doubt, increase the width of the corresponding side of the chest; but its influence on the results of mensuration is vague and but slightly marked, because the normal difference of size between the two sides, which is a variable quantity, must be taken into account. M. Woillez is of opinion, however, that an excess of two and a half or three centimètres may justly be accounted pathological. He is, so far as we know, the first who has investigated by mensuration the dilating action of enlarged spleen or tympanitic bowels on the thorax. He has very clearly shown that both do produce such an effect, and, what is interesting, that the expansion caused by the former will be clearly ascertainable by mensuration, when inspection discovers no irregularity of form.

We have next an elaborate article on Pleuritic Effusion. Its most interesting feature consists in the proof it gives that, in the outset of pleurisy with effusion, the affected side is not, and cannot be, dilated. This is established by the help of the physiological propositions we have already dwelt on. At the earliest period of pleurisy, the effused liquid is exposed to the action of two antagonist powers. One of these is the concentric force or aspiration of the lung, which tends to draw the fluid over the entire pulmonary surface; the other is gravity, which tends to cause its accumulation at the depending parts. This latter force is at first either wholly or in a great measure overcome by the former; but, in proportion as the effusion increases, the law of gravity acquires the supremacy. The consequence is, that the liquid at first spreads over every part of the lung, and has no clearly defined upper border. During this period dilatation cannot take place, because aspiration still exists in the pleura. The second period commences as soon as the concentric force of the lung is exhausted; the fluid now gravitates to the lower part of the chest, and dilatation commences. As M. Woillez has given no calculation of the mean duration of the first period, we do not think he is quite justifiable in denying the possibility of dilatation from a pleurisy of three hours' existence, and in assuming the presence of a physiological heteromorphism in the cases where such an occurrence is stated by Laennec to have taken place. The third period is that of absorption of the effused fluid, with or without retraction of the affected side. The doctrine here advanced in explanation of the absence of dilatation in the first stage of pleuritic effusion is, to our minds, much more satisfactory than that set forth by Dr. Stokes. That pathologist conceives that paralysis of the intercostals is a necessary condition for the dilatation of these muscles, and has founded on this view an explanation, of which we willingly acknowledge the ingenuity, while we must question its probable stability. If his theory be sound, dilatation, as he himself admits, should not occur, unless in cases where inflammatory action, with diminished innervation of the muscular wall, had preceded. Now, the united experience of the most distinguished observers (if we except only Dr. Stokes himself) proves that the dilatation of emphysema is at once *costal* and *intercostal;* yet it surely will not be maintained that the muscles are in that disease affected in the manner alluded to. Besides, as was stated in a former Number, we cannot concede to Dr. Stokes that intercostal paralysis is by any means a constant occurrence in pleurisy.

The special characters of dilated thorax from pleuritic effusion are examined at great length; and those of general retraction investigated with equal care. M. Woillez regards the following peculiarities as fundamental properties, if we may so speak, of the latter species of deformity: 1. Fixed inclination of the ribs downwards. 2. Irregularity of the contracted side. 3. Depression of the shoulder of that side. 4. Lowering of the corresponding nipple. 5. Lateral deviation of the dorsal spine towards the healthy side. The importance of these distinctive marks does not require to be insisted on. But our author has also detected the frequent occurrence, in consequence of pleurisy, of limited and local retraction; a phenomenon which, we believe, had scarcely been noticed by previous observers. He enumerates five points of the chest at which he has met with this species of deformity. The following table exhibits its relative frequency, as well as that of general retraction:

| Different species of Retraction. | Right side. Pleurisy, (10 cases.) | Right side. Pleuro-pneumonia, (5 cases.) | Left side. Pleurisy, (9 cases.) | Left side. Pleuro-pneumonia, (3 cases.) | Total, (27 cases.) |
|---|---|---|---|---|---|
| General retraction...... | 1 | 0 | 0 | 0 | 1 |
| Partial: anterior.............. | 4 | 2 | 0 | 0 | 6 |
| Partial: antero-lateral.. ... | 1 | 0 | 0 | 0 | 1 |
| Partial: antero-posterior... | 1 | 0 | 0 | 0 | 1 |
| Partial: posterior...... .... | 1 | 0 | 3 | 1 | 5 |
| Partial: postero-lateral.... | 0 | 0 | 1 | 0 | 1 |
| | 8 | 2 | 4 | 1 | 15 |

The phenomena of double and of partial or circumscribed pleurisy furnish M. Woillez with no very remarkable inferences.

In the chapter on Pericarditic Effusion we find the important intimation that, in thirty-two cases of pericarditis, observed by M. Louis in the space of four years, elevation of the precordial region existed in every instance but one. It is no doubt possible that, in some subjects, the heteromorphism may have been either partially or wholly physiological; but its extreme regularity of occurrence shows its importance as a diagnostic sign. We do not, however, observe in these pages any novel information respecting it. Their author assumes that absorption of fluid effused into the pericardium cannot be attended with any retraction of the chest. "The heart," he urges, "is not a compressible organ, like the lung; and the false membranes organized on its surface cannot act on it, as the pulmonary pseudo-membranes do on the lung; that is, they cannot keep its volume reduced so as to prevent its advance to the walls of the chest, in proportion as absorption proceeds." Perhaps a depression of the precordial region, he continues, may be observed in individuals who have recovered from pericarditis; but such deformity should be looked on as the effect of concomitant pleurisy. This is plausible, and certainly not easily refuted: nevertheless, we do not wholly admit its correctness, and await the evidence of observation.

M. Woillez next passes to the influence of adventitious productions on the shape of the thorax; but confines his remarks to the effects of tubercle, the only species he had occasion to observe. He measured accurately the circular extent of the two sides of the chest in forty-one phthisical subjects, and found its mean capacity less by 4·7 centimètres than that of individuals free from pectoral disease. But the inference apparently deducible from this estimate is not exactly that which it really affords. In fact, the low average capacity did not depend on each individual tuberculous patient having a smaller thorax than healthy persons, (ten among them, that is, one fourth of their number, had chests of greater circular extent than the physiological standard,) but upon the extremely small pectoral development of fifteen among them. M. Woillez shall give his own conclusion from this and some other related facts.

"Narrowness of the chest does not cause a predisposition to phthisis, unless it be coupled with an incomplete development of the transverse diameter. It is therefore incorrect to say that subjects of small thoracic capacity are more liable than others to pulmonary tubercles. If the transverse diameter bear a fitting ratio to the remaining ones, the predisposition to the complaint is not stronger than in full-chested individuals. But I should add, that a few subjects, whose chest is below the standard in lateral extent, escape the disease, when that imperfect general configuration depends on insufficient length of the ribs." (p. 484.)

Our author did not discover any heteromorphism which could be ascribed either to the accumulation of tubercles, or to the supposed contraction of the chest induced by the cicatrization of tuberculous cavities.

Before taking leave of M. Woillez, we have a few general remarks to offer. It is easy to perceive, even from the analysis which our limited space has allowed us to give of them, that its physiological section promises results of greater novelty and importance than the pathological division of the work really supplies. This is however, in a great measure, to be attributed to the comparatively small number of cases of each disease its author chanced to meet with in the course of his labours. It does not, in the smallest degree, lessen the value of such enquiries; for, as the least reflection will show, the heteromorphisms which pathologists were in the habit of assigning to given diseases could not legitimately be so assigned until they had been subjected to such investigation as they have now had at the hands of M. Woillez. He has laid the foundation whereon others may securely build. The practical importance of his researches will, we feel convinced, be many a time acknowledged at the bedside of the patient, by all physicians who are emulous of the high character of philosophic observers. The zeal with which he has laboured in the cause of truth; the patient perseverance with which he has borne the detestable task of working out their medical history from persons who have no interest in communicating it, (M. Woillez held no official appointment in the hospitals where he observed,) deserve grateful acknowledgment on our parts. He is, we are pleased to find, quite free from the dictatorial air that lessens the pleasure felt in studying most of the productions of the Medical Society of Observation. There is a happy union of the firmness of tone which the extent, originality, and method of his researches entitle him to assume, with the modesty becoming his youth and previous want of reputation. Nor does he seem to us to fall into a very common fault with those whose researches are in any degree original, that of overrating their importance. Unlike his countryman, M. Piorry, "le père de la percussion médiate," as he is fond of styling himself, and who would almost discard auscultation entirely, and give undivided empire to the plessimeter,—he everywhere regards inspection and mensuration as only secondary instruments for the discovery of pulmonary disease. The complexity of the details in the book renders its study a laborious task: this labour might, we think, be materially lessened by the construction of a general table, giving at a single view the substance of the work and the relations of its various parts. We would likewise suggest to M. Woillez, if the public call on him for a second edition, the propriety of altering his mode of using the words *physiological* and *pathological*, already adverted to.

